# Editorial: Parsing Psychology: Statistical and Computational Methods Using Physiological, Behavioral, Social, and Cognitive Data

**DOI:** 10.3389/fpsyg.2019.02694

**Published:** 2019-11-29

**Authors:** Jason C. Immekus, Pietro Cipresso

**Affiliations:** ^1^Educational Leadership, Evaluation and Organizational Development, University of Louisville, Louisville, KY, United States; ^2^Department of Psychology, Universitá Cattolica del Sacro Cuore, Milan, Italy; ^3^Applied Technology for Neuro-Psychology Lab, IRCCS Istituto Auxologico Italiano, Milan, Italy

**Keywords:** machine learning, quantitative methods, psychological data science, computational methods, statistical methods and models

Advancements in statistical and computational methods have much to contribute to promoting new discoveries in psychology. In particular, as found in diverse disciplines as marketing, health care, and medicine, machine learning and big data offer similarly unique potential to inform, test, and advance our knowledge of mental processes, brain functioning, and behavior in ways that were not previously possible. Psychological data science represents the integration of data science into psychology to explore the abundant information provided by physiological, behavioral, social, and cognitive data alone and together. The merging of psychology and data science is due to a host of factors, including, for example, more readily accessible data, cheaper and more powerful computational processing, and larger data storage capacity. As a result, social science researchers are well-positioned in their ability to incorporate data science into their work to advance and promote our understanding of key factors associated with important psychological outcomes (e.g., mental health).

This Research Topic represents a collection of papers that demonstrate the use, or potential, of psychological data science to pursue both hypothesis-driven experiments and bottom-up data exploration through modern statistical and computational models. As these studies illustrate, machine learning algorithms are already expanding our knowledge of key factors associated with designated outcomes (e.g., academic and brain-based disorders) or have the potential to build on existing and emerging areas of research (e.g., scale development). Broadly, the papers comprising this Research Topic fall into three general areas as related to the application of data science in psychological and psychometric research, including: Supervised and Unsupervised Machine Learning Computing, Contemporary Psychometrics, and Emerging Areas of Research.

## Supervised and Unsupervised Machine Learning Computing

Machine learning represents a branch of artificial intelligence in which statistical model building is based on automatically implemented algorithms to identify data patterns for classification and prediction purposes. While there are several categories of machine learning, supervised, and unsupervised learning are, at this time, most commonly found in psychological research. Specifically, supervised learning encompasses the development of statistical models in which user-specified inputs (e.g., predictor variables) and outputs (e.g., outcome variables) are provided, and include classification (e.g., decision tree) and regression (e.g., ordinary least squares and elastic net) techniques. On the other hand, unsupervised learning includes algorithms in which only input data (e.g., variables) are analyzed, with the aim of identifying meaningful patterns in the data, such as cluster analysis (e.g., *K*-means) and dimensionality reduction (e.g., principal components analysis) techniques.

The papers within this section demonstrate the use of supervised and unsupervised learning procedures to address a range of substantive topics in psychological research. Specificity, Zhao et al. apply unsupervised clustering methods using static and dynamic resting-state functional magnetic resonance imaging to four different datasets for the classification of different brain-based disorders (e.g., Alzheimer's Disease and Post-Traumatic Stress Disorder) to compare diagnosis based on traditional approaches that rely on clinical interviews and behavioral assessments. In another study, Guixeres et al. compared two types of neural networks, Multi-layer Perceptron and Radial Basis Function, to determine the use of three neurophysiological measures (i.e., brain response, heart rate variability, and eye tracking) to predict the effectiveness of an online advertisement within an online video platform (i.e., YouTube). Situated in a dynamic network framework, Hasmi et al. apply network models using multilevel time-lagged regression to examine if genetic liability to psychopathology and childhood trauma relate to the network structure of various emotions (e.g., cheerful and relaxed), based on real-time data collected on pairs of sibling and twins using the experience sampling method (ESM; i.e., structured diary technique to record emotions). In consideration of the use of big data to understand how individuals use of technology to ways that lead to positive engagement and peace,  Guadagno et al. present a theoretical framework for standardizing Peace Data (e.g., group identity) based on hypothetical and real-data. Specifically, real-data was subjected to a social network analysis to examine gender differences (e.g., connections and likes) in social media use within the context of a large financial institution over a 6-month period.

Additional studies applied machine learning within educational settings that have implications to psychological and educational assessment. Based on TIMSS 2011 data, Yoo uses elastic net to identify a prediction model of Korean 4th graders mathematics achievement using 162 teacher and student variables, in which 12 student and 5 teacher variables are identified as significant predictors. The study also examines the process of scale development from a machine learning perspective. Polyak et al.'s innovative research in computational psychometrics demonstrate the use of machine learning to assess middle school students' collaborative problem-solving (CPS) sub-skills (e.g., evaluate and maintaining a shared understanding), based on their performance within a virtual, online game in which the player must collaborate with a virtual “agent” with the information to “win” the game. Uniquely, this research demonstrates the ways in which traditional test theory practices can be integrated into assessment design and machine learning algorithms to develop psychometric models to establish classification and measurement rules to assess important student traits.

## Contemporary Psychometrics

Studies within this section demonstrate the contribution of current and emerging psychometric analyses to the development and validation of instruments to assess a range of individual traits. In general, the psychometric models and analyses used across the majority of studies have a long, rich history in instrument development (e.g., factor analysis). Their application within these studies represent efforts to address key literature gaps in the assessment of clinically important traits, including the utility of virtual reality to promote the ecological validity of neuropsychological testing. Collectively, the papers serve to highlight the contribution of psychological data science to instrument design and validation.

The papers in this section illustrate the use of contemporary psychometrics and technology to advance our assessment of psychological constructs. For instance, Maldonato et al.'s research focuses on the development of a reduced version of the Temperament and Character Inventory for use among clinical samples, and using logistic regression to examine the predictive validity of scores to determine the presence/absence of psychiatric Axis pathologies. In response to lack of available measures, Olivencia-Carrión et al. present their work on the development and validation, via confirmatory factor analysis, of a measure of mobile phone abuse among young Spanish-speakers in Spain. Vallejo et al. use exploratory factor analysis to examine the dimensionality of the Perceived Stress Scale and, subsequently, examine perceived stress throughout three European countries (Great Britain, France, Spain), based on a large dataset (*N* = 37,451) obtained from a smoking cessation program. To promote the ecological assessment of visual attention, Gamito et al.'s pilot study demonstrates the rich potential of virtual reality technology as an alternative to noted limitations of traditionally administered neuropsychological tests. Verhagen et al.'s study focuses on the assessment of momentary reward-related Quality of Life, based on data collected via ESM among individuals with severe mental illness, which they use to generate a momentary rQoL statistic and, second, conduct a Monte Carlo simulation study to determine four outcomes (e.g., required sample size to reliably measure individuals' behavior setting). Last, Provenzi et al.'s paper presents efforts to develop an observational approach to measure emotional stress regulation among preschool aged children in which they identify and describe the theoretical and methodological considerations in the selection of stress-related episodes.

## Emerging Areas of Research

The papers comprised in the previous sections demonstrate the integration of computational and statistical techniques into psychological and psychometric research. Contrary, the papers in this section address substantive topics in psychological research that provide a foundation for future research based on the application of psychological data science. In particular, the common thread across the subsequent studies is the rich potential that technology affords in data collection and analysis, as well as intervention delivery.

The studies represented in this section provide a foundation for subsequent research that relies heavily on the efficiency of technology in practice (e.g., counseling) and research to promote individuals' psychological well-being. Specifically, Connors and Rende's paper points to the potential of automatic coding to assess how individuals' physical and mental activities are related to subsequent decision-making through Movement Pattern Analysis. Zaytseva et al.'s  paper discusses how establishing the link between errors (e.g., sequential and proximity) in cognitive test performance and brain networks may contribute to understanding the association between cognitive functioning and brain circuits and subsequent task performance among individuals with schizophrenia. This study, in particular, illustrates how psychological data science can pull together diverse types of data (e.g., cognitive tests and fMRI) to identify and understand the connections between brain networks and cognitive task performance to determine, as the authors' state, “the common denominators of the generalized deficits.” Vitale et al. used a randomized crossover design to examine the effects of chronotype (i.e., circadian rhythmicity) on mood state (e.g., depression and anger) and perceived physical exertion following acute high intensity interval exercise. Based on prior research on the effects of online psychosocial intervention for engaging youth in mental health treatment, D'Alfonso et al. detail the development of the moderated online social therapy (MOST) project designed as an online peer support for youth experiencing mental health issues (e.g., depression). In particular, their work explores the ways in which computational and artificial intelligence methods (e.g., Chatbots) may offer a mechanism to use automated user-specific therapy to supplement and enhance support delivered to patients by real-life moderators (and clinicians). Finally, Anwar et al. offer an informative book review on *Networks of the Brain*, authored by Olaf Sporns, which focuses on the application of network science in neuroanatomy. As presented in the review, the book offers several lines of inquiry for psychological data science regarding the data types that can contribute to modeling the brain within a complex network approach.

The papers included in this Research Topic illustrate the potential of psychological data science to unlock the wealth of information provided by diverse data types (e.g., physiological, social, and cognitive). The access to affordable, efficient processing is providing rich opportunities to implement a range of statistical and computational models and estimation procedures that were not readily available for use in applied research within the past few decades. Consequently, the rise of data science and big data is directly influencing the practice and research across a range of disciplines (e.g., marketing and medicine), including psychology. As such, the papers included in this Research Topic offer only a sampling of the body of work in the emerging discipline of psychological data science. Moving forward, we very much look forward to the ways in which data science and big data further develop the knowledge base in psychology.

## Author Contributions

JI and PC contributed to the writing of the editorial and review of the corresponding Research Topic papers.

### Conflict of Interest

The authors declare that the research was conducted in the absence of any commercial or financial relationships that could be construed as a potential conflict of interest.

